# Accuracy analysis of the new artificial anatomical marker positioning method (shoulder-to-shoulder) in preventing leg length discrepancy in total hip arthroplasty

**DOI:** 10.3389/fsurg.2024.1487716

**Published:** 2024-12-19

**Authors:** Wang Ze-feng, Fang Yang-zhen, Zheng Yong-qiang, Lin Zhen-yu, Lin Liang, Liu Xiao-feng, Zhang Chi, Zhang Jin-shan

**Affiliations:** Department of Orthopedics, Jinjiang Municipal Hospital/Clinical Research Center for Orthopaedic Trauma and Reconstruction of Fujian Province, Jinjiang, Quanzhou, Fujian, China

**Keywords:** anatomical marker, hip arthroplasty, leg length discrepancy, control, artificial intelligence

## Abstract

**Objective:**

By comparing the hip arthroplasty parameters planned with the AIHIP three-dimensional simulation surgery system, this study analyzes the accuracy of the new femoral-side “shoulder-to-shoulder” artificial anatomical marker positioning method in femoral-side prosthesis implantation and the prevention of leg length discrepancy in hip arthroplasty.

**Methods:**

A retrospective collection of 47 patients who underwent initial total hip arthroplasty at our hospital from August 2020 to December 2022 and met the inclusion and exclusion criteria was used as the study subjects. The average age was 67.34 ± 10.86 years (32–80 years), including 17 males and 30 females; 25 cases on the left side and 22 cases on the right side. According to the Garden classification for fractures: 4 cases of type II, 4 cases of type III, and 21 cases of type IV; according to the ARCO staging for femoral head necrosis: 1 case of stage III and 6 cases of stage IV; according to the Crowe classification: 2 cases of type I and 3 cases of type II; according to the K-L grading: 2 cases of stage III and 4 cases of stage IV. The postoperative pelvic anteroposterior x-ray measurement parameters and prosthesis model results guided by the new “shoulder-to-shoulder” artificial anatomical marker positioning method (“shoulder-to-shoulder” group) were compared with the corresponding parameter results planned by the AIHIP three-dimensional simulation surgery system (AIHIP simulation surgery group). All postoperative pelvic anteroposterior x-ray measurement parameters were corrected according to the radiographic magnification, and the differences in bilateral lower limb length, tip-to-shoulder distance, and osteotomy distance between the two groups were compared. The paired *t*-test was used to compare the differences in bilateral lower limb length, tip-to-shoulder distance, and osteotomy distance; descriptive analysis was used to evaluate the consistency of prosthesis model matching.alpha = 0.05 (both sides).

**Results:**

The differences in bilateral lower limb length for the “shoulder-to-shoulder” group and the AIHIP simulation surgery group were 1.07 ± 1.18 mm and 1.28 ± 2.41 mm, respectively, with a difference of −0.28 ± 2.16 mm between the two groups. The paired *t*-test results showed no statistically significant difference (*P* = 0.508). The tip-to-shoulder distance and osteotomy distance for the “shoulder-to-shoulder” group were 15.93 ± 2.96 mm and 7.81 ± 2.73 mm, respectively, while the corresponding parameters for the AIHIP simulation surgery group were 17.70 ± 3.39 mm and 9.21 ± 4.05 mm. The differences in tip-to-shoulder distance and osteotomy distance between the “shoulder-to-shoulder” group and the AIHIP simulation surgery group were −1.78 ± 2.54 mm and −1.22 ± 3.17 mm, respectively. The paired *t*-test results showed statistically significant differences in the comparison of tip-to-shoulder distance and osteotomy distance between the two groups (both *P* < 0.01). The matching rates of acetabular and femoral prosthesis models were 91.48% and 95.74%, respectively.

**Conclusion:**

The new artificial anatomical marker positioning method (shoulder-to-shoulder) and the AIHIP three-dimensional simulation surgery method show good consistency in preventing leg length discrepancy in hip arthroplasty. This proves that using this method can accurately implant the femoral-side prosthesis during surgery and prevent postoperative leg length discrepancy.

## 1 Introduction

Total hip arthroplasty (THA) is one of the most effective treatments for end-stage hip disease. Precise matching of the prosthesis during surgery is crucial for achieving ideal mechanical transmission of the lower limbs and maintaining long-term stability of the hip joint, as well as preventing prosthesis dislocation, loosening, failure, leg length discrepancy, and a series of associated complications (1, 2). Many orthopedic surgeons have conducted extensive research and efforts in accurately implanting the prosthesis and preventing leg length discrepancy, including the combined use of preoperative accurate assessment, intraoperative positioning markers, and intraoperative testing methods. Preoperative templating plays an important guiding role in predicting prosthesis size, position, rotation center, and controlling leg length discrepancy. Traditional two-dimensional preoperative templating methods are widely used in clinical practice due to their simplicity and low cost, but their accuracy is relatively low (3, 4). With the development of technology, the use of digital templates for preoperative measurement has gradually gained certain advantages and promotion (5). Preoperative measurement using electronic devices to measure radiographic data and estimate prosthesis placement and size has higher accuracy and reliability (6–8). There are also CT-based three-dimensional planning software, which have high accuracy but are time-consuming and complex to operate (9). In recent years, the rapid development of artificial intelligence-based three-dimensional planning software systems, such as the domestically developed AIHIP system, has been notable. Studies have shown that the three-dimensional simulation surgery planning of the AIHIP system is significantly superior to x-ray-based two-dimensional planning in terms of prosthesis placement accuracy, clinical outcomes, and radiographic results, making it an ideal standard for THA prosthesis implantation (10–13). However, since the system is still in the third-party development and promotion stage, and it is based on the patient's CT three-dimensional data, preoperative CT scans need to be completed, and the scan data must be exported, stored, and sent to a third-party platform for preoperative planning, which takes time and has many uncertainties. For hip diseases, especially hip fractures, the surgical timing is short. According to the latest 2022 AAOS evidence-based clinical practice guidelines for the management of hip fractures in older adults (14), it is recommended that hip fracture surgery be performed within 24–48 h to achieve better functional outcomes. For these reasons, the author currently uses the new femoral-side “shoulder-to-shoulder” artificial anatomical marker positioning method for two-dimensional digital template preoperative planning and intraoperative guidance for prosthesis implantation in THA. The studies in the first three parts have shown that this method can effectively prevent postoperative leg length discrepancy. In the author's previous research, the accuracy of the new “shoulder-to-shoulder” artificial anatomical marker positioning method was compared with the contralateral comparison method and the Shuck test method in measuring leg length during hip arthroplasty for femoral neck fractures. The results showed that the femoral “shoulder-to-shoulder” anatomical marker positioning method can simply, effectively, and accurately reduce postoperative leg length discrepancy in elderly patients undergoing THA for femoral neck fractures (15). Taking advantage of the free policy during the promotion stage of the AIHIP system, the author will import the preoperative CT scan data of patients using the new “shoulder-to-shoulder” artificial anatomical marker positioning method into the AIHIP system for simulation surgery planning, generating an ideal postoperative reference model. The postoperative x-ray measurement parameters and prosthesis model results guided by the new “shoulder-to-shoulder” artificial anatomical marker positioning method will be compared with the corresponding parameters from the AIHIP system simulation surgery, aiming to further verify the accuracy of this method in femoral-side prosthesis implantation and prevention of postoperative leg length discrepancy during hip arthroplasty.

## 2 Materials and methods

### 2.1 Case selection

#### 2.2.1 Inclusion criteria

Patients undergoing initial unilateral THA surgery using the new “shoulder-to-shoulder” artificial anatomical positioning method due to:

(1) Unilateral femoral neck fracture (Garden II-IV)
(2) Unilateral femoral head necrosis (ARCO III or IV)
(3) Unilateral hip dysplasia (Crowe I or II)
(4) Unilateral osteoarthritis of the hip due to degenerative changes (K-L III or IV)

Preoperative planning and surgery were both completed by the same chief surgeon.

#### 2.2.2 Exclusion criteria

(1) Deformities affecting accurate measurement due to trauma or surgery on the operative limb.
(2) Shortening or anatomical variations in the contralateral limb, or previous hip arthroplasty or internal fixation surgery affecting bilateral comparison.
(3) Hip dysplasia (Crowe III or IV).
(4) Lack of complete medical records (including the prosthesis model used during surgery) or radiographic images (including preoperative and postoperative anteroposterior pelvic radiographs, preoperative pelvic CT scans).

### 2.2 Clinical data

This study is a retrospective study aimed at verifying the accuracy and effectiveness of the new “shoulder-to-shoulder” artificial anatomical marker positioning method in guiding THA results, using the ideal parameter results of the AIHIP system simulated THA surgery as the reference standard. A total of 197 patients who underwent initial unilateral THA at our hospital from August 2020 to December 2022 were retrospectively collected, and 47 patients who met the inclusion and exclusion criteria were included as study subjects. The average age was 67.34 ± 10.86 years (32–80 years), including 17 males and 30 females; 25 cases on the left side and 22 cases on the right side. According to the Garden classification for fractures: 4 cases of type II, 4 cases of type III, and 21 cases of type IV; according to the ARCO staging for femoral head necrosis: 1 case of stage III and 6 cases of stage IV; according to the Crowe classification for hip dysplasia: 2 cases of type I and 3 cases of type II; according to the K-L grading for senile degenerative osteoarthritis of the hip: 2 cases of stage III and 4 cases of stage IV (Table 1). All 47 patients were implanted with prostheses from DePuy, USA, including 47 Pinnacle acetabular cups; 1 Tri-Lock high-offset stem, 1 Tri-Lock standard stem, 2 Summit standard stems, 39 Corail standard stems, and 4 Corail collared stems. The results of all 47 patients generated by the two different methods were divided into two groups, namely the “shoulder-to-shoulder” group and the AIHIP simulation surgery group.

(1) “Shoulder-to-Shoulder” Group: The postoperative pelvic anteroposterior x-ray measurement parameters and actual prosthesis models of all 47 patients were used as the observation group.
(2) AIHIP Simulation Surgery Group: The corresponding parameter results and ideal prosthesis models from the AIHIP three-dimensional simulation surgery planning based on the preoperative pelvic CT scan data of all 47 patients were used as the control group.

**Table 1:** General information of patients (*n* = 47, cases).

Age, years (mean ± SD)	Height, mm (mean ± SD)	Weight, kg (mean ± SD)	Sex		Left and right edge		Garden fracture classification			ARCO classification		Crowe classification		K-L classification	
			Male	Female	L	R	II	III	IV	III	IV	I	II	III	IV
67.34 ± 10.86	158.96 ± 17.35	65.54 ± 18.42	17	30	25	22	4	4	21	1	6	2	3	2	4

### 2.3 “Shoulder to shoulder” group treatment method

**Figure 1:**
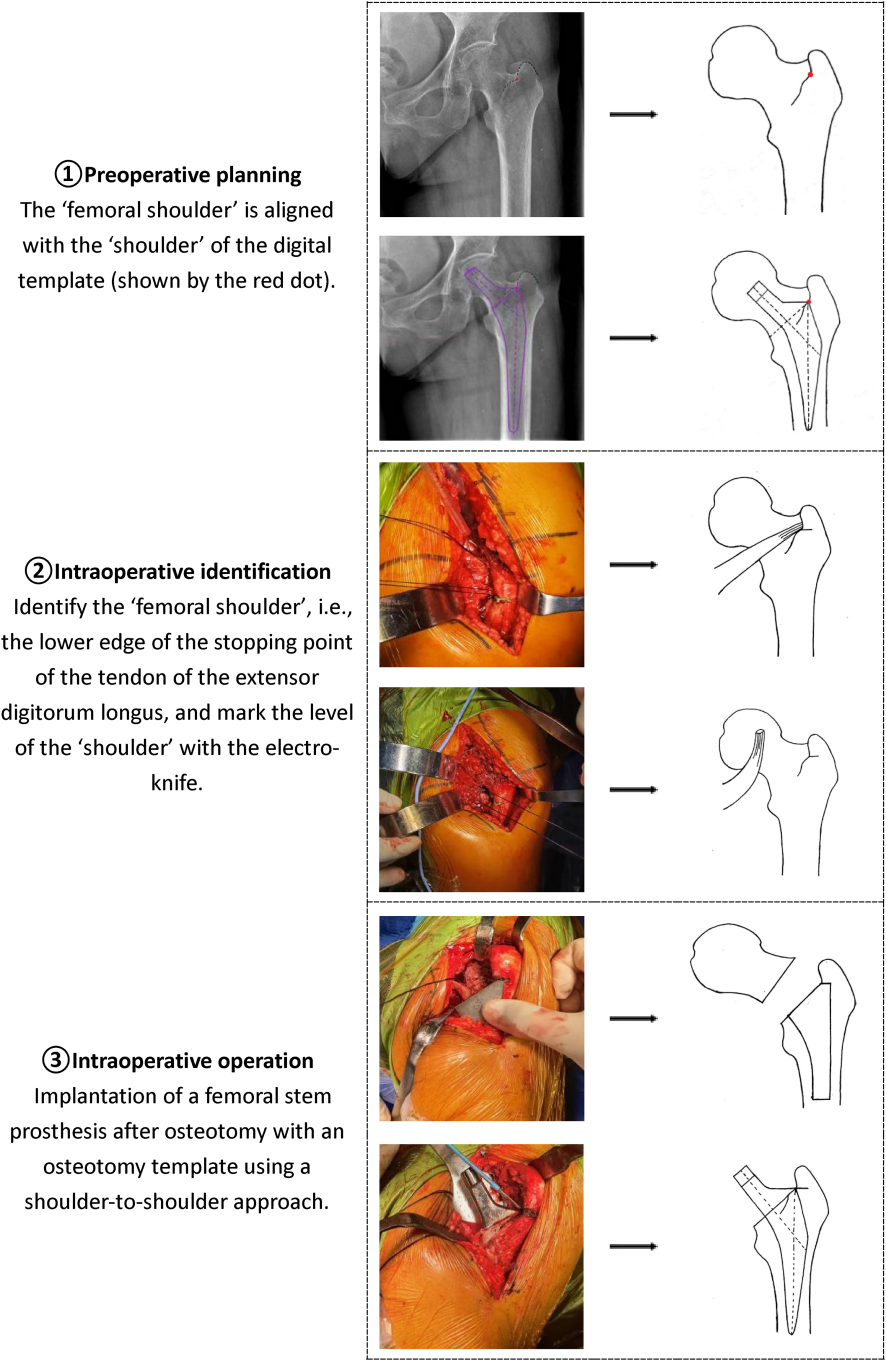
Schematic diagram of the new artificial anatomical marker mositioning method (shoulder-to-shoulder).

(1) Radiographic Examination Preoperative standard anteroposterior pelvic x-ray requirements:
  1. The patient should be in a supine position with both lower limbs extended, keeping the hip and knee joints in a straight neutral position. Both feet should be internally rotated by 10–15° to better counteract the anteversion angle of the femoral neck and accurately project the true length and neck-shaft angle of the femoral neck (16).
  2. The imaging range should include the hip joint, proximal femur, pubis, ischium, and ilium.
  3. There should be no projection deformation of the femoral neck.
  4. The bone texture of the hip joint should be clear and sharp, and the ischial spine should be clearly visible (11).
(2) Preoperative Planning with the “Shoulder-to-Shoulder” Method Using Two-Dimensional Digital Templates After taking a standard anteroposterior pelvic x-ray, the x-ray image is imported into the Smart Joint 2.0 two-dimensional mobile planning platform software for two-dimensional digital template measurement. The accurate scale length is input, and the most suitable template model is selected to match the acetabulum and femur, aiming to restore the length of the operative limb and the femoral offset as much as possible. The required acetabular cup prosthesis and femoral stem prosthesis types and models are recorded, and the appropriate head length (long, standard, short) is adjusted intraoperatively based on the actual situation. The specific measurement methods are as follows:
  1. Draw a horizontal reference line through the lower edges of the teardrops and the ischial tuberosities on both sides. Mark the bottom of the teardrop, the ilioischial line, and the superolateral edge of the acetabulum on the x-ray image.
  2. Determine the size of the acetabular cup. Adjust the digital template of the cup prosthesis to an abduction angle of 40°±5°, placing the lower boundary of the cup at the same level as the teardrop line, with the medial boundary close to the ilioischial line or the lateral side of the teardrop, ensuring sufficient bone coverage (>75%) and that the outer edge does not excessively protrude beyond the true acetabular edge.
  3. First, determine the position of the “femoral shoulder” on the anteroposterior pelvic x-ray. Select an appropriately sized femoral stem and overlap the femoral stem template at the appropriate intramedullary position, ensuring that the “stem shoulder” aligns with the “femoral shoulder” and that the stem aligns with the medullary canal axis. Determine the ideal level of the femoral head rotation center based on the neck-shaft angle, usually at or near the level of the greater trochanter tip (above the greater trochanter tip level in patients with hip valgus; below the greater trochanter tip level in patients with hip varus). The part of the femoral stem template inserted into the femoral medullary canal should achieve optimal contact with the endosteal cortex of the proximal femur.
  4. The femoral offset should be restored as close to normal as possible. The template should generally be placed in the middle range of the femoral neck to allow for intraoperative adjustment to long, standard, or short head lengths based on testing.
(3) Perform THA using the novel “Shoulder-to-Shoulder” anatomical marker positioning method (Figure 1), while documenting the actual sizes of the prostheses used during the procedure.
(4) Postoperative Review Take standard anteroposterior pelvic x-rays postoperatively, following the same requirements as preoperative imaging. Compare the relevant evaluation parameters measured from the postoperative x-rays and the actual prosthesis model and size used during surgery with the corresponding results from the AIHIP group.

### 2.4 AIHIP simulated operation group

(1) Radiographic Examination

Preoperative multi-slice spiral pelvic CT scan requirements:

1. The patient should be in a supine position.
2. The scanning range should include from the upper edge of the pelvis to 15 cm below the lesser trochanter of the femur.
3. The pitch should be ≤1, with a reconstruction matrix of 512*512 or higher, and an inter-slice distance of less than 1 mm.

(2) AIHIP System Simulation Surgery

The thin-slice CT scan data of the pelvis is output and stored in DICOM format, and imported into the AIHIP three-dimensional planning software system (AIHIP system; Beijing Changmu Valley Medical Technology Co., Ltd.). The system uses algorithms to automatically remove impurities and perform three-dimensional reconstruction, displaying the bone structure accurately in axial, coronal, and sagittal views linked with the original CT scan images. The system's neural network intelligently segments the acetabulum and femur, achieving artificial intelligence segmentation of the acetabulum and femoral head. The algorithm then completely separates the pelvis and femur, allowing clear observation of the femoral head and acetabulum morphology. The AIHIP system can utilize an automatic search engine based on databases and deep learning to identify relevant anatomical locations, including the anterior superior iliac spine of the pelvis, pubic symphysis, lesser trochanter, and greater trochanter. It automatically calculates parameters such as acetabular diameter, femoral medullary cavity diameter, and neck-shaft angle, and corrects the pelvis and lower limbs to a neutral position based on the pelvic anterior plane formed by the bilateral anterior superior iliac spines and pubic symphysis. Based on the identified anatomical locations, it intelligently calculates the preoperative femoral offset, combined offset, and leg length discrepancy, providing references for the surgeon. After completing the initial correction of the pelvis, the acetabular cup prosthesis is placed at an abduction angle of 40° and anteversion angle of 20° according to the corrected pelvic coordinate system. The system displays the contour of the imported prosthesis on the CT image and calculates and displays the bone coverage rate of the acetabular cup prosthesis in real-time. The surgeon selects the type of femoral stem prosthesis to be used (e.g., Corail, Summit, Tri-Lock), and the software intelligently matches the optimal femoral stem model from the corresponding prosthesis database based on the femoral medullary cavity diameter. It automatically matches with the three-dimensional model, simulates the prosthesis placement, simulates femoral neck osteotomy, determines the vertical distance from the osteotomy line to the upper edge of the lesser trochanter, and after placing the prosthesis, automatically matches with the three-dimensional model and selects the femoral head size based on the acetabular cup size. After completing the simulation placement of the femoral-side prosthesis, the system calculates and displays the bone coverage rate of the acetabular cup prosthesis in real-time based on the overlap rate of the prosthesis and bone. It intelligently calculates the optimal positions of the acetabular and femoral prostheses, plans the optimal results, and provides an ideal reference model for prosthesis type and component size. It can also automatically calculate the difference in vertical distance from the inner edge of the bilateral lesser trochanters to the teardrop line (leg length discrepancy), combined offset difference, vertical distance from the tip of the greater trochanter to the shoulder of the femoral stem (tip-to-shoulder distance), and vertical distance from the upper edge of the lesser trochanter to the osteotomy plane (osteotomy distance). Since the AIHIP three-dimensional simulation surgery system planning is based on CT scan data, its correction is automatic, and the simulated three-dimensional image is a standard 1:1 scale image. The system's calculated leg length discrepancy, combined offset difference, tip-to-shoulder distance, osteotomy distance, and prosthesis model are actual values and models. The ideal parameters and prosthesis model size obtained from the AIHIP three-dimensional simulation surgery are compared with the corresponding results of the “shoulder-to-shoulder” group.

### 2.5 Intergroup evaluation indicators and measurement methods

The postoperative anteroposterior pelvic x-rays were marked and measured by two trained and experienced orthopedic attending physicians using the same computer's Picture Archiving and Communication System (PACS). The average of the measurement results was taken. The relevant evaluation indicator data automatically calculated by the AIHIP three-dimensional simulation surgery system were provided by Beijing Changmu Valley Medical Technology Co., Ltd.

(1) Radiographic Magnification Factor: By measuring the diameter of the prosthetic head on the postoperative anteroposterior pelvic x-ray and comparing it with the actual diameter of the prosthetic head used during surgery, the radiographic magnification factor is calculated. The formula is: Magnification Factor=Diameter of Prosthetic Head on Postoperative Pelvic x-ray/Actual Diameter of Prosthetic Head Used During Surgery.
(2) Radiographic Bilateral Lower Limb Length Discrepancy: Measure the vertical distance from the inner edge of the lesser trochanter to the teardrop line on both sides to represent the radiographic bilateral lower limb length. Divide the measured data by the radiographic magnification factor to obtain the actual radiographic lower limb length. The difference between the two sides is the actual bilateral lower limb length discrepancy, which is then compared with the bilateral lower limb length discrepancy automatically calculated by the AIHIP three-dimensional simulation surgery system.
(3) Tip-to-Shoulder Distance: Measure the vertical distance from the tip of the greater trochanter to the shoulder of the femoral stem. Divide the measured data by the radiographic magnification factor to obtain the actual tip-to-shoulder distance, and compare it with the tip-to-shoulder distance automatically calculated by the AIHIP three-dimensional simulation surgery system.
(4) Osteotomy Distance: Measure the vertical distance from the upper edge of the lesser trochanter to the osteotomy plane. Divide the measured data by the radiographic magnification factor to obtain the actual osteotomy distance, and compare it with the osteotomy distance automatically calculated by the AIHIP three-dimensional simulation surgery system.

Prosthesis Model Consistency Evaluation: Observe and compare the differences between the actual prosthesis model used during surgery and the prosthesis model planned by the AIHIP three-dimensional simulation surgery system.

### 2.6 Statistical methods

The measurement data for radiographic bilateral lower limb length discrepancy, tip-to-shoulder distance, and osteotomy distance conform to a normal distribution and are expressed as mean ± standard deviation (SD). Intergroup comparisons are performed using paired *t*-tests. The consistency evaluation of prosthesis model matching is conducted using descriptive analysis. SPSS 26.0 (IBM Corp) is used for the analysis. The significance level is set at alpha = 0.05 (two-sided).

## 3 Results

### 3.1 Comparison of lower limb length difference (leg length difference) between the two groups

**Figure 2:**
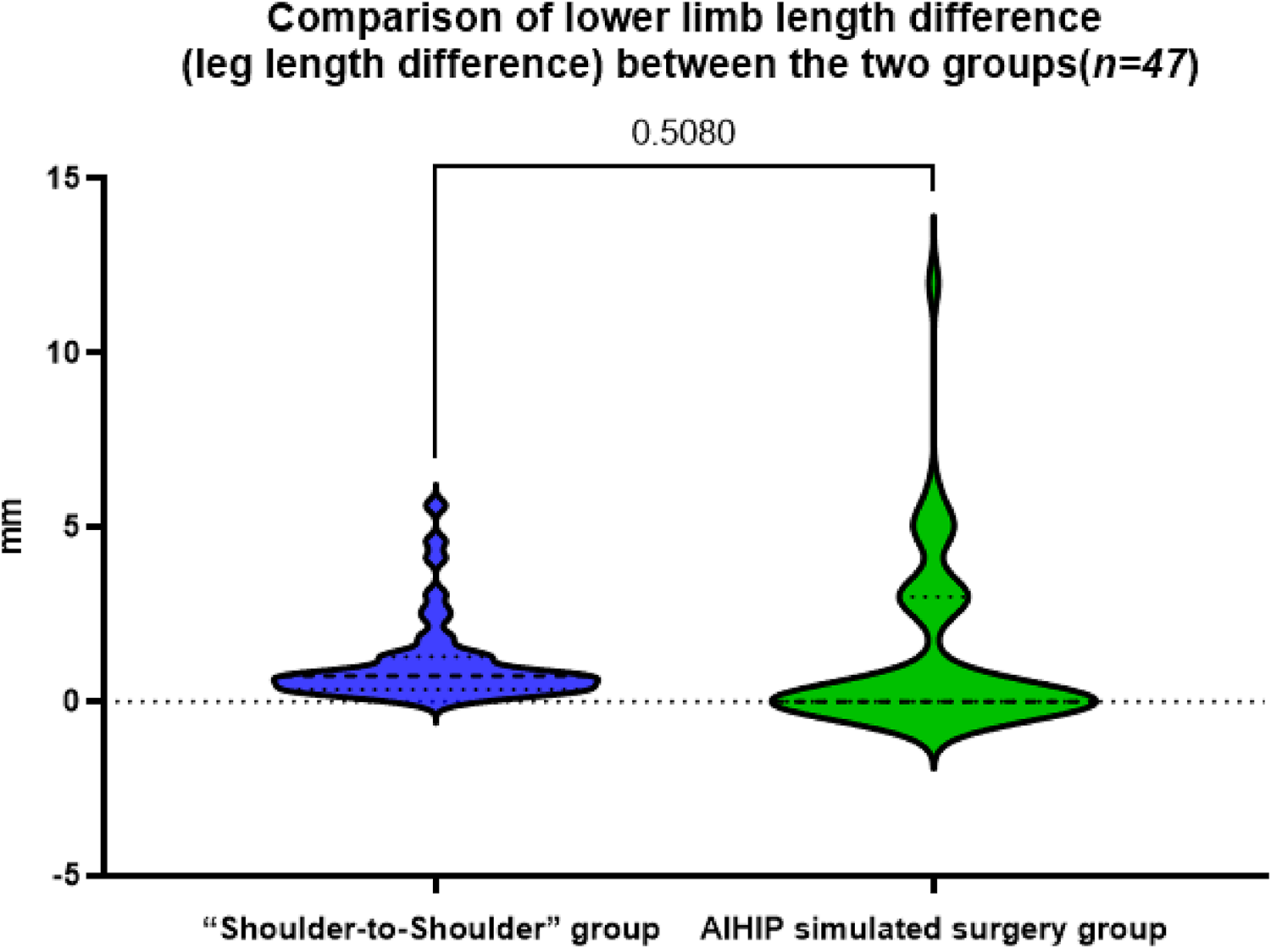
Comparison of the difference in bilateral lower limb lengths between the “Shoulder-to-shoulder” group and the AIHIP simulated surgery group, *P* = 0.5080 > 0.05, indicating no statistically significant difference.

The actual difference of lower limb length in the “shoulder-to-shoulder” group was 1.07 ± 1.18 mm, the difference of lower limb length in the AIHIP simulated surgery group was 1.28 ± 2.41 mm, and the difference of lower limb length between the “shoulder-to-shoulder” group and the AIHIP simulated surgery group was −0.28 ± 2.16 mm (95%CI: −0.41, 0.81), and the paired *T*-test showed no statistically significant difference (*P* = 0.508) (Figure 2). There was no statistically significant difference in the comparison of bilateral lower limb length differences between the two groups, indicating that the new “Shoulder-to-Shoulder” anatomical marking method can achieve an ideal lower limb length difference similar to the AIHIP three-dimensional simulated surgery planning results. Moreover, the limb length difference was controlled within 5 mm, providing patients with a more ideal and satisfactory lower limb length.

### 3.2 Comparison of the acromion distance between the “shoulder-to-shoulder”

**Figure 3:**
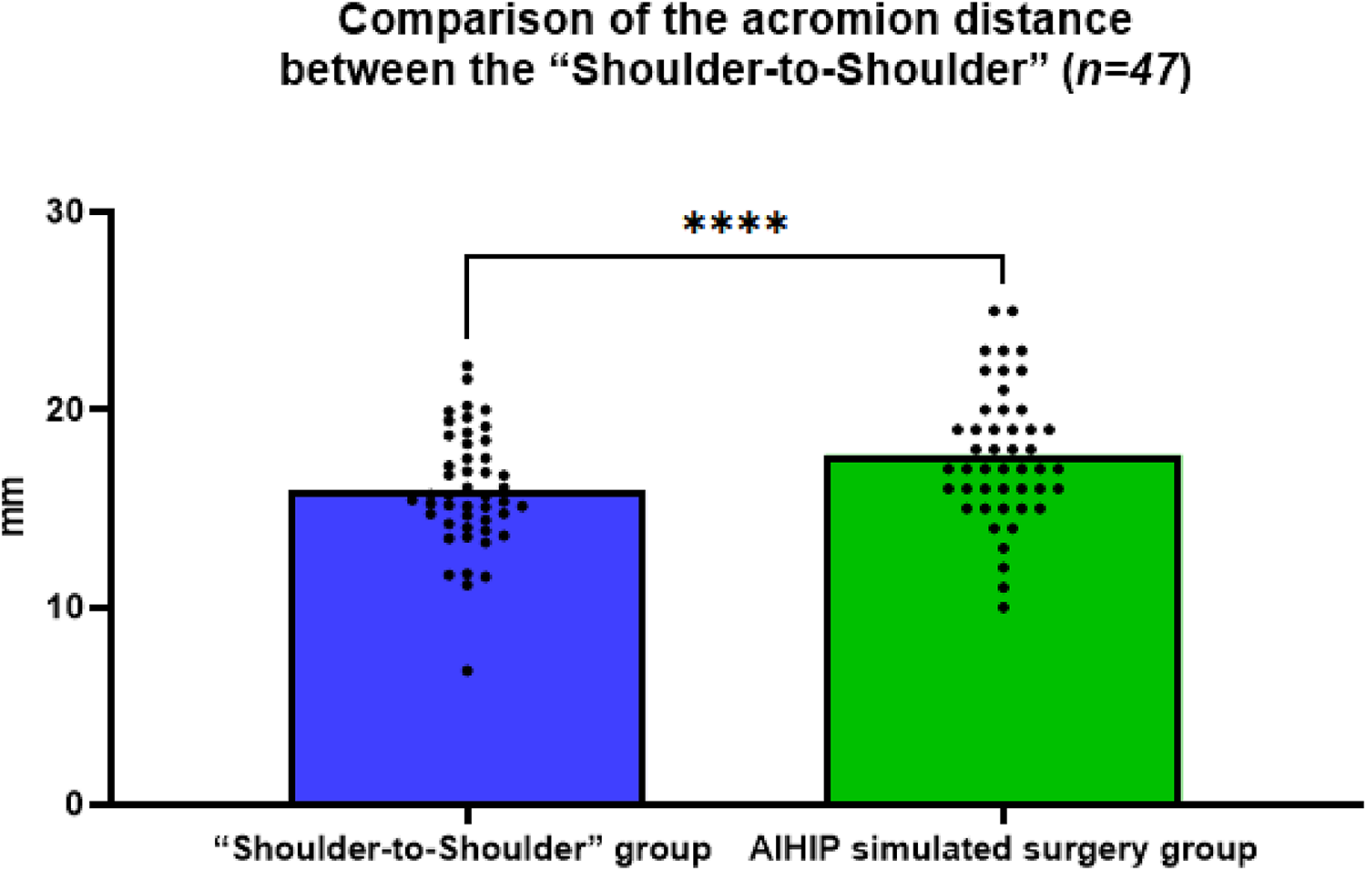
Comparison of the acromion distance between the “Shoulder-to-shoulder” group and the AIHIP simulated surgery group, ****P < 0.0001, indicating a statistically significant difference.

The actual acromion distance in the “Shoulder-to-Shoulder” group was 15.93 ± 2.96 mm, while in the AIHIP simulated surgery group it was 17.70 ± 3.39 mm. The difference in acromion distance between the “Shoulder-to-Shoulder” group and the AIHIP simulated surgery group was −1.78 ± 2.54 mm (95% CI: −2.52, −1.02). The paired *t*-test results showed a statistically significant difference (*P* < 0.0001) (Figure 3), with an average difference of 2 mm between the two groups, suggesting that the reference to the length of the highest point of the greater trochanter distance in the AIHIP surgical planning to guide the depth of prosthesis implantation is questionable.

### 3.3 Comparison of osteotomy distance between the two groups

**Figure 4:**
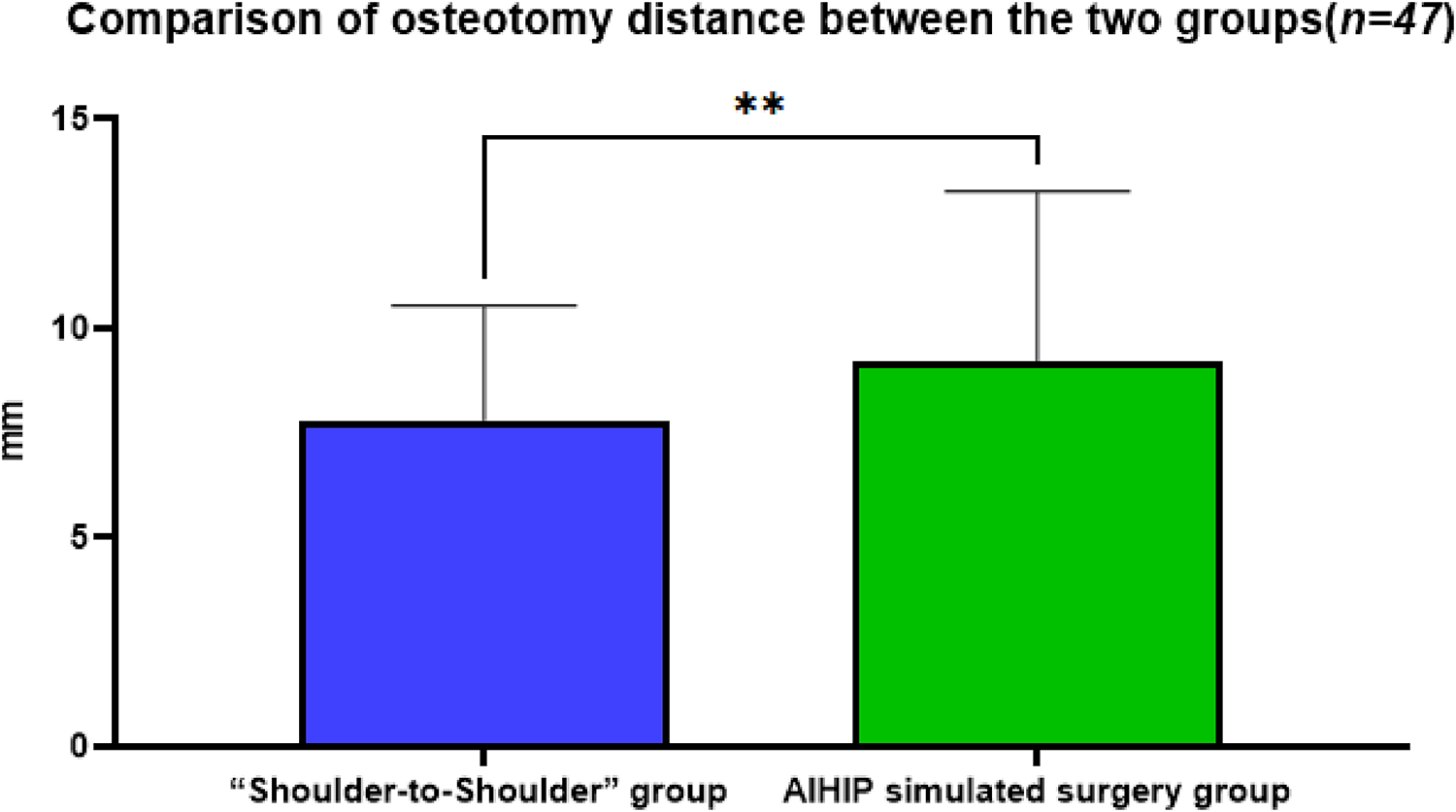
Comparison of the osteotomy distance between the “Shoulder-to-shoulder” group and the AIHIP simulated surgery group, *P* = 0.0032 < 0.01, indicating a statistically significant difference.

The actual osteotomy distance in the “Shoulder-to-Shoulder” group was 7.81 ± 2.73 mm, while in the AIHIP simulated surgery group it was 9.21 ± 4.05 mm. The difference in osteotomy distance between the “Shoulder-to-Shoulder” group and the AIHIP simulated surgery group was −1.22 ± 3.17 mm (95% CI: −2.31, −0.50). The paired *t*-test results showed a statistically significant difference in the comparison of osteotomy distances between the two groups (*P* < 0.01) (Figure 4), with an average difference of about 1.4 mm. The mean difference in this data is small, and whether it can be used for intraoperative reference depends on the surgical access and intraoperative specifics.

### 3.4 Consistency evaluation of pseudobody size between two groups

Compared to the preoperative planning of the AIHIP system, the actual acetabular prosthesis model used during surgery was the same in 24 cases (51.06%), differed by one size in 19 cases (40.43%), and differed by more than one size in 4 cases (8.51%). The matching rate between the actual acetabular prosthesis model used during surgery and the AIHIP system's preoperative planning was 91.48%. For the femoral prosthesis, the model was the same in 27 cases (57.45%), differed by one size in 18 cases (38.30%), and differed by more than one size in 2 cases (4.26%). The matching rate between the actual femoral prosthesis model used during surgery and the AIHIP system's preoperative planning was 95.74% (Table 2). This indicates that the AIHIP three-dimensional simulated surgery system has high accuracy in predicting the actual prosthesis models used during surgery and can be used to guide the use of prosthesis models during surgery.

**Table 2:** Matching rate between the actual prosthesis model used during surgery and the AIHIP system's simulated surgery prosthesis model.

	Difference between the actual model used and the AIHIP system plan model (size)			The matching rate between the planned size and the actual size^a^
	0	1	>1	
Acetabular side	24	19	4	91.48%
Femur side	27	18	2	95.74%

^a^ If the size of the model is the same or the difference is in one model, the model is considered to be matching.

## 4 Discussion

THA is one of the common surgeries in orthopedic surgery. The artificial hip joint includes acetabular, femoral, and femoral head prosthesis components. Inaccurate placement, size, and type selection of prosthesis components can cause serious postoperative complications such as unequal leg length, joint instability and dislocation, periprosthetic fractures, and even prosthesis failure. Detailed preoperative planning and precise intraoperative operations can accurately predict the appropriate prosthesis type and size and implant it in the correct position, reducing intraoperative and postoperative complications, and achieving an ideal biomechanical and functional artificial hip joint. In previous studies on cadaver specimens and clinical applications, the authors used a new femoral “Shoulder-to-Shoulder” anatomical marking method to guide preoperative planning and intraoperative prosthesis positioning. It was observed that satisfactory bilateral lower limb lengths could be achieved both in cadaver specimens and in patients with femoral neck fractures and hip osteoarthritis in clinical applications. This study compares the radiographic evaluation indicators of postoperative pelvic anteroposterior films collected in previous clinical trials with the ideal indicators after the AIHIP system planning correction and prosthesis implantation to evaluate and verify the accuracy of the anatomical marking method used by the authors.

For orthopedic surgeons, the goal of preoperative planning for THA is to reconstruct hip joint function while maximizing the restoration of equal bilateral lower limb lengths. Reviewing previous relevant research conclusions from abroad, it is recommended that the combined offset difference and bilateral lower limb length difference be controlled within 5 mm or 6 mm after THA. This can effectively reduce the wear of the artificial joint prosthesis, avoid gluteus medius weakness, and improve hip joint mobility and lower limb gait (17–20).

The AIHIP three-dimensional simulated surgery system can comprehensively analyze the hip joint anatomical structure of patients undergoing THA, perform detailed comparative analysis of the pre-used artificial prosthesis types, and consider them holistically. Compared with previous preoperative designs, the prosthesis matching is more precise, theoretically reducing postoperative complications such as unequal limb lengths.

Since the tip of the greater trochanter is an anatomical structure that is easily exposed and identified during THA, it is often used by orthopedic surgeons to assist in evaluating the level of the rotational center and balancing the length of the lower limbs (21, 22), or as a reference mark for judging the depth of prosthesis implantation. During THA, the authors also focused on the relative position of the greater trochanter and the implanted femoral stem prosthesis. After using the “Shoulder-to-Shoulder” anatomical marking method to implant the femoral prosthesis, the vertical distance from the tip of the greater trochanter to the “shoulder” of the femoral stem was measured.

In this study, the average acromion distance measured in the “Shoulder-to-Shoulder” group was 15.93 ± 2.96 mm, while the AIHIP simulated surgery group gave an acromion distance of 17.70 ± 3.39 mm. Statistical analysis showed a significant difference between the two groups in the comparison of acromion distances, with an average difference of less than 2 mm. This indicates that the depth of prosthesis implantation can refer to the acromion distance length provided by the AIHIP three-dimensional simulated surgery planning. However, it is difficult to use this measurement accurately during surgery because the tip of the greater trochanter is covered with periosteum and other soft tissues of a certain thickness. The periosteum and attached soft tissues at the highest point of the greater trochanter must be completely removed during surgery to truly apply the acromion distance length given by the AIHIP three-dimensional simulated surgery planning. This would cause more damage and reconstruction difficulties for the patient and would not guarantee early postoperative hip joint function (23). Therefore, some scholars suggest that it is difficult to accurately execute the anatomical landmark positioning of the highest point of the greater trochanter to determine the osteotomy level during surgery (24).

Studies have shown that measuring and accurately osteotomizing the femoral osteotomy distance (the distance from the osteotomy plane to the upper edge of the lesser trochanter) can reduce unequal bilateral lower limb lengths after THA (25). In this study, the “femoral shoulder” was first identified as the anatomical marker for osteotomy during THA. The proximal end of the osteotomy template was placed at the marked “femoral shoulder,” and the osteotomy line was marked on the femoral neck according to the osteotomy template for accurate osteotomy. This method does not consider the reserved length of the osteotomy distance during surgery, as it depends on the type of prosthesis selected. The cases included in this study used three different types of femoral stem prostheses: Tri-Lock, Summit, and Corail. The osteotomy template for Corail is 45°, while the osteotomy templates for Summit and Tri-Lock are 50°, resulting in different final osteotomy distances.

Comparing the osteotomy distances with the AIHIP three-dimensional simulated surgery planning, the actual osteotomy distance in the “Shoulder-to-Shoulder” group was 7.81 ± 2.73 mm, while in the AIHIP simulated surgery group it was 9.21 ± 4.05 mm. Statistical analysis showed a significant difference between the two groups in the comparison of osteotomy distances, with an average difference of about 1.4 mm. Although the difference is statistically significant, the average difference is not large. Based on the authors’ extensive experience with THA, especially in the posterolateral approach, it is challenging to accurately determine the specific position of the upper edge of the lesser trochanter during surgery. Measuring the distance from the upper edge of the lesser trochanter to the osteotomy plane is difficult and has low accuracy.

The AIHIP three-dimensional simulated surgery planning is significantly superior to the two-dimensional preoperative planning based on x-rays in terms of prosthesis model accuracy, clinical outcomes, and radiographic results. It can serve as an ideal standard for the implantation of THA models (10–13). This study further evaluated the consistency between the actual prosthesis models used during surgery guided by this marking method and the ideal prosthesis models planned by the AIHIP three-dimensional simulated surgery system. According to the AIHIP three-dimensional simulated surgery system's prosthesis consistency evaluation criteria, if the actual implanted model during surgery is the same as or differs by one size from the AIHIP predicted model, the predicted model is considered matched. In this study, compared to the AIHIP three-dimensional simulated surgery system's planned acetabular prosthesis models, 43 cases (91.48%) had the same or one size difference; for femoral prosthesis models, 45 cases (95.74%) had the same or one size difference. This indicates that the AIHIP three-dimensional simulated surgery system has high accuracy in predicting the actual prosthesis models used during surgery and can be used to guide the use of prosthesis models during surgery.

However, the AIHIP three-dimensional simulated surgery system's planned prosthesis types are based on the surgeon's pre-selected femoral stem prosthesis types (such as Corail, Summit, Tri-Lock, etc.). The software then intelligently matches the optimal femoral stem model specifications from the corresponding type prosthesis database. Currently, it cannot intelligently identify individual differences such as femoral neck-shaft angle and femoral neck length to match the appropriate femoral stem type, and it has not yet achieved fully autonomous, automatic, and intelligent matching of prosthesis types and models.

In this study, we evaluated the accuracy of the “shoulder-to-shoulder” anatomical marker positioning method across various hip joint pathologies, including femoral neck fractures, hip osteoarthritis, and congenital hip dysplasia (types I and II). Our findings indicate that the accuracy of this method is minimally impacted in patients with femoral neck fractures. This is attributed to the restoration of the fundamental anatomical relationship between the femur and acetabulum, which can be effectively achieved intraoperatively through precise alignment and imaging guidance.

In patients with hip osteoarthritis, although preoperative anatomical variations or bone destruction on the acetabular side can occur, our study included patients with relatively mild variations or damage. Thus, the method demonstrated substantial accuracy in these cases. Patients with severe variations, particularly those classified as Crowe types III and IV, were excluded from this study due to their potential impact on postoperative limb length stability following THA. Consequently, in the studied cohort, the longitudinal displacement of the rotational center was minimal, resulting in limited and controlled influence on postoperative outcomes and overall accuracy.

For patients with congenital hip dysplasia (types I and II), the method maintained moderate accuracy due to the relatively predictable bone morphology, supported by detailed preoperative and intraoperative imaging assessments. In summary, while the method's application exhibits some variability across different pathological conditions, its accuracy remains relatively unaffected in cases with mild anatomical variations, thereby demonstrating its practical value in specific clinical settings.

### 4.1 Limitations of this study

(1) The study mainly focused on the prevention of unequal lower limb lengths, collecting only parameters related to lower limb length. It did not further collect and compare parameters such as offset distance and acetabular prosthesis placement angle.
(2) Due to the need for thin-slice CT scans, patients are exposed to relatively increased radiation, and the economic cost is correspondingly higher. Additionally, doctors need to separately upload CT scan data and the pre-selected prosthesis types, which consumes a certain amount of time.

## 5 Conclusions

The new anatomical marking method (Shoulder-to-Shoulder) and the AIHIP three-dimensional simulated surgery method show good consistency in preventing unequal bilateral lower limb lengths in THA. This demonstrates that using this method can accurately implant the femoral prosthesis during surgery and prevent postoperative unequal lower limb lengths.

## Data Availability

The raw data supporting the conclusions of this article will be made available by the authors, without undue reservation.
